# Facilitating community participation in family planning and contraceptive services provision and uptake: community and health provider perspectives

**DOI:** 10.1186/s12978-020-00968-x

**Published:** 2020-08-08

**Authors:** Adam Silumbwe, Theresa Nkole, Margarate N. Munakampe, Joanna Paula Cordero, Cecilia Milford, Joseph Mumba Zulu, Petrus S. Steyn

**Affiliations:** 1 Department of Health Policy and Management, School of Public Health, University of Zambia, P.O Box 50110, Lusaka, Zambia; 2 Department of Obstetrics and Gynaecology, University Teaching Hospital, Lusaka, Zambia; 3 UNDP-UNFPA-UNICEF-WHO-World Bank Special Programme of Research, Development and Research Training in Human Reproduction (HRP) and Department of Sexual and Reproductive Health and Research (SRH), Geneva, World Health Organization, Geneva, Switzerland; 4 MRU (MatCH Research Unit), Department of Obstetrics and Gynaecology, Faculty of Health Sciences, University of the Witwatersrand, Durban, South Africa; 5 Department of Health Promotion, School of Public Health, University of Zambia, Lusaka, Zambia

**Keywords:** Community, Participation, Family planning, Contraceptives, Programs, Accountability, Trust, Strategies, Motivation

## Abstract

**Background:**

Although community participation has been identified as being important for improved and sustained health outcomes, designing and successfully implementing it in large scale public health programmes, including family planning and contraceptive (FP/C) service provision, remains challenging. Zambian participants in a multi-country project (the UPTAKE project) took part in the development of an intervention involving community and healthcare provider participation in FP/C services provision and uptake. This study reports key thematic areas identified by the study participants as critical to facilitating community participation in this intervention.

**Methods:**

This was an exploratory qualitative research study, conducted in Kabwe District, Central Province, in 2017. Twelve focus group discussions were conducted with community members (*n* = 114), two with healthcare providers (*n* = 19), and ten in-depth interviews with key community and health sector stakeholders. Data were analyzed using a thematic analysis approach.

**Results:**

Four thematic categories were identified by the participants as critical to facilitating community participation in FP/C services. Firstly, accountability in the recruitment of community participants and incorporation of community feedback in FP/C.

programming. Secondly, engagement of existing community resources and structures in FP/C services provision. Thirdly, building trust in FP/C methods/services through credible community-based distributors and promotion of appropriate FP/C methods/services. Fourthly, promoting strategies that address structural failures, such as the feminisation of FP/C services and the lack FP/C services that are responsive to adolescent needs.

**Conclusions:**

Understanding and considering community members’ and healthcare providers’ views regarding contextualized and locally relevant participatory approaches, facilitators and challenges to participation, could improve the design, implementation and success of participatory public health programmes, including FP/C.

## Plain English summary

Community participation remains a fundamental principle within the primary healthcare policy. Its association with improved and sustained health outcomes within communities still makes it a key focus of various public health initiatives. However, challenges remain on how to design and successfully implement community participation in public health programmes, including in family planning and contraceptive (FP/C) services in various settings. In this qualitative study, we explore Zambian study participants’ perceptions of community participation and identify factors that can facilitate its application in FP/C service provision. Twelve focus group discussions were conducted with community members (*n* = 114) and two with healthcare providers (*n* = 19). Ten in-depth interviews were held with key community and health sector stakeholders. Data were analysed using thematic analysis. The results show that four thematic categories are critical in facilitating community participation in FP/Cs services provision. Firstly, accountability in the recruitment of community members and incorporation of community feedback in FP/C programming. Secondly, engagement of existing community resources and structures in FP/C services provision. Thirdly, building trust in FP/C methods/services through credible community-based distributors and promotion of appropriate FP/C methods/services. Fourthly, promoting strategies that address structural failures such as the feminisation of FP/C services and the lack of FP/C services responsive to adolescent needs. Understanding and considering community members’ and healthcare providers’ views regarding contextualized and locally relevant participatory approaches, facilitators to and challenges to participation, could improve the design, implementation and success of participatory public health programmes, including FP/C.

## Background

Community participation has been recognised as the foundation strategy for Primary Health Care services [1]. It provides a platform for communities to be involved in both activities and decisions that shape their health. Some of the benefits of community participation, such as community empowerment, are not only critical for enhanced acceptability and uptake of healthcare interventions but also addressing health inequalities [2]. However, incorporating community participation into health service delivery programmes remains a challenge due to the complexity of participatory processes, who participates and the inherent power relations in various contexts [3]. Socio-cultural factors that directly influence individual tendencies, including lack of awareness, discouraging perceptions about participation outcomes, and the lack of confidence in the participatory process, are equally challenging to achieving community participation [4].

Family planning and contraceptive (FP/C) programmes recommend community participation as a key strategy for improved service provision [5]. This is partly because uptake of FP/C methods and services is shaped by several socially embedded community factors such as religious values, political climate and dominating moral understandings [6]. Key policies such as the Family Planning 2020 (FP 2020), note that community participation is vital in expanding access to information, services and supplies to women and girls in remote communities [7]. The FP 2020 also highlights the role of community participation in holding governments accountable for providing quality reproductive health services [8]. The World Health Organization’s (WHO) Guideline on ensuring human rights in the provision of contraceptive information and services [9], as well as the Global strategy for women’s, children’s and adolescents’ health [10], equally emphasize the role of community participation in increasing met needs and improving utilisation of FP/C services.

Although community participation is widely advocated for at the policy level, designing and successfully implementing participatory FP/C programmes remains a challenge in most settings [11]. Achieving community participation in promoting and accessing reproductive health services, including contraceptives, implies identifying community members who truly represent community needs, understanding what constitutes a community in a particular context, as well as, unpacking the contextual power relations [12]. A recent scoping review found that community participation continues to be inadequately addressed in large-scale FP/C programmes [13]. Community and healthcare providers’ unequal power relationships tend to be a barrier to successful participation in FP/C services, i.e., unaligned priorities and the inability of community members to communicate their needs, and health professionals not being receptive. Ensuring the best possible ways to facilitate the involvement of local communities is, therefore, a critical question in the attainment of global FP/C goals.

This study was part of the formative phase of a multi-country project (UPTAKE) conducted in Zambia, Kenya and South Africa, which sought to increase met needs for FP/C, through a participatory intervention involving community and healthcare providers (HCPs) in FP/C service provision. The specific aim of the formative phase was to develop an approach to engage the health system, HCPs and community actors in FP/C services provision. In this paper, we explore Zambian study participants’ perceptions of how to facilitate community participation in FP/C service provision.

The unmet need for FP/C methods and services among married women in Zambia is 21%, of which 14% are spacers and 7% are limiters [10]. Unmet need for modern contraceptive methods is significantly higher among rural to rural migrant women (OR 1.30, 95%CI 1.00–1.70 *p* < 0.05) and rural non-migrant women (OR 1.41, 95%CI 1.06–1.85 *p* < 0.01) compared to urban non-migrant women [14]. The contraceptive prevalence rate is 47%, with noticeable differences between rural and urban communities [10]. Given the context, understanding processes and strategies to facilitate local involvement, therefore, becomes crucial in efforts to improve FP/C service provision and uptake in Zambia.

## Methods

### Study design

The present study focuses on the exploratory qualitative research conducted during the UPTAKE project formative phase in Zambia. The UPTAKE project was a complex-designed intervention, aiming to increase contraceptive met needs, through community and healthcare provider participation in the provision and use of FP/C methods and services, within a human-rights framework [15]. The exploratory qualitative research not only contributed to the development of the intervention, but also to the identification of key human-rights framework domains, within which to contextualize activities to increase met needs [9]. In this manuscript, we specifically explored community participation practices and activities according community and healthcare provider perspectives. Other exploratory qualitative research activities are published elsewhere [15–18].

### Study setting

In Zambia, the exploratory qualitative research was conducted in Kabwe District, Central Province, which has a population of 217,843 of which 58,381 (26.8%) are women of reproductive age (15–49 years old) [19]. The Central Province was chosen based on its high rate of unmet need for FP/C services, second only to Luapula Province in Zambia [19]. Kabwe District, the provincial capital, has a total of 38 health facilities, ranging from the general hospital, clinics and primary healthcare centres. The district health management team oversees all these facilities. Katondwe health facility was chosen as the study site because it has a large catchment area catering to both rural and urban communities (peri-urban). This facility served as a recruitment point for the community focus group discussions.

### Data collection

Fourteen (14) focus group discussions (FGDs), each lasting between 60 to 90 min, were conducted. Of these, 12 consisted of community members (FP/C users and non-users), and two were conducted with HCPs stratified as managerial and frontline providers (Table 1). The FGDs not only provided community and health system perspectives but also allowed different user categories to discuss specific issues in the provision and use of FP/C services. To minimise convenient responses and promote freedom among community participants, the HCPs were not present during the community FGDs. Ten (10) in-depth interviews (IDIs), lasting between 30 and 60 min, were conducted with various key stakeholders– community leaders and providers of FP/C services (Table 2). The IDIs provided perspectives on the possible role of key stakeholders in participatory activities.

**Table 1:** FGD participants

Focus group discussion categories	Age in years	Participants
**Community members**		
Females, urban, adolescents	15–19	10
Females, rural, adolescents	15–19	09
Females, urban young adults	20–34	08
Females, rural young adults	20–34	10
Females, urban adults	35–49	08
Females, rural adults	35–49	09
Females, unmarried young adults	20–34	10
Females, married young adults	20–34	10
Females, no-children	18–49	10
Males, adolescents	15–19	10
Males, young adults	20–34	10
Males, adults	35–49	10
**Healthcare providers**		
Healthcare providers-managerial	–	10
Healthcare providers-frontline	–	09
**Total participants**		**133**

**Table 2:** IDI participants

Participants (categories)	Interviews
Political leader (ward councillor)	1
NHC	1
NGO-SRH	1
Traditional leader (headman)	1
District health office	1
Provincial medical office	2
Secondary school teacher	1
Religious leader	2
**Total**	**10**

FGD and IDI guides explored what constitutes community participation, who should participate, strategies and challenges of the community collaborating with health providers in FP/C services provision. Discussions were audio-recorded in the local language, Bemba, with participants’ permission, then transcribed and translated verbatim into English. The data collection team consisted of experienced data collectors with a good understanding of community participation dynamics. The FGDs were conducted at the health facility, while IDIs were held at locations that were convenient to the key stakeholders over three months.

### Study participants and recruitment

Male and female community members, consisting of FP/C users and potential users within the reproductive age range 15–49 years, were purposively sampled for the FGDs. They were categorised according to age, rural vs. urban residence, marital status and parity (Table 1), to ensure a variation of information sources. The age categories (*n =* 9) consisted of adolescents (15–19 years), younger adults (20–34 years) and older adults (35–49 years). Female groups were further categorised according to their location (*n* = 6) – either rural or urban, marital status (*n* = 2) – either married or unmarried and according to parity (*n* = 1) – with and without children. The recruitment process of community members followed a participatory approach by engaging the Kabwe District Health Office (DHO) through a local district coordinator. The coordinator worked with the nursing sister-in-charge at Katondwe health facility, together with the neighbourhood health committees (NHC), in recruiting the different categories of community members for the FGDs. Local actors were also involved in identifying community members to participate in the interviews. The consenting process and final recruitment of study participants were done by the research team (without the local actors being present) to facilitate valid informed consent and minimise selection bias.

Similarly, HCPs were purposively sampled from Katondwe and other healthcare facilities to ensure a wide representation of FP/C service providers in the district. The DHO invited FP/C service providers from all the facilities in Kabwe district. The recruitment of key stakeholders was purposively guided by the DHO, particularly those that were most active in FP/C services in the district (Table 2). Some of the key stakeholders in FP/C services included political, traditional and religious leaders, sexual and reproductive health (SRH) non-governmental organisation representatives, district and provincial medical officers, a teacher from the education sector and an NHC representative.

### Data analysis

Thematic analysis was adopted using the qualitative data analysis software, NVivo version 10 (QSR International), to organise and manage the data across the three countries [20]. The data analysis process started with reflections on the information gathered through field notes, memos and observations. A coding framework was developed from preliminary data and iteratively modified after every coding activity by the qualitative research teams from the three countries. Once the transcripts from the three countries were transcribed verbatim and imported to NVivo, further coding at country-level was conducted to identify emergent themes (Table 3). A systematic approach involving identifying, checking and iterations of codes during the entire data analysis process ensued across the three countries to facilitate reliable and accurate data analysis [21]. Once the coding activity was completed, the reports consisting of country-level broader and analytical codes from NVivo were further analysed.

**Table 3:** Data code-list

Major themes	Emergent themes
Accountability in the recruitment of community participants and in FP/C services provision	▪ Defining the context of participation ▪ Defining who participates in FP/C services ▪ Incorporation of community feedback in FP/C services programming
Engagement of community structures/resources in FP/C services provision	▪ Involvement of family planning champions ▪ Leveraging established community structures ▪ Motivation of community members ▪ Capacity building of FP/C services counselling among volunteers
Building trust in FP/C methods/Services	▪ Promotion of appropriate FP/C methods/services ▪ Community and health provider meetings/dialogue ▪ Ensuring credibility of community-based distributors
Facilitative strategies	▪ Defeminisation of FP/C services ▪ Health facilities responsive towards the delivery of adolescent FP/C services

### Ethical considerations

This study received WHO Ethics Review Committee (ERC) and Research Project Review Panel (RP2) approval (A65896). Ethical approval for the Zambian research activities was also obtained from the University of Zambia Biomedical Research Ethics Committee (UNZABREC). All participants (> 18 years) provided written informed consent to participate in the study. Participants under the age of 18 years provided written assent, and their guardians provided written consent for their participation. If participants could not read and write, a thumbprint was taken, and a witness was required to be present during the consenting process, and sign consent on their behalf. The participants gave separate consent to being audio recorded.

## Results

Data are presented based on community members’, healthcare providers’ and key stakeholders’ perspectives. Through the data analysing process, four core thematic categories emerged as critical to facilitating community participation in FP/C services. These include accountability, community engagement, building trust and facilitative strategies. Though this study explored perspectives from a varied group of participants, no major differences in perspectives according to participant category were reported with regards to community participation in FP/C services.

1. Accountability in the recruitment of community participants and incorporation of community feedback in family planning and contraceptive services provision

Accountability was thought to be an important factor in facilitating community participation in FP/C services provision. Both the community members and HCPs indicated that to facilitate community participation, FP/C services programmes had to be accountable in the way they defined participation, recruited community participants, as well as embraced community feedback. Community participation was defined as the willingness to be or the process of being involved in activities that improved the lives and health of communities. It was a combination of community member efforts in activities and programmes of mutual benefit. Community members expressed that participatory programmes were beneficial if they facilitated knowledge, skills and resource sharing. Additionally, they expressed that meaningful community participation in FP/C services could only be attained if community members were adequately sensitized about the programme before implementation.

*“I think community participation … this is the willingness of the community to participate in all the activities that are taking place in our centres. Since we are talking about family planning, it means they should involve themselves in sensitizing people especially to those who have knowledge about it. They should take part in sensitizing those people who don’t have knowledge about family planning, that’s what I think.” [Female FGD, Unmarried, UZFG_C008].*

The community members narrated that people were more likely to be encouraged to participate in FP/C services when they felt the right beneficiaries were engaged. They suggested some possible participants in FP/C services activities. They indicated that both adults and adolescents should be involved because FP/C is a cross-cutting issue.

*“I think it’s everyone who should participate. Both adults and young people because these family planning issues affects all of us.” [Female FGD, Urban Adolescent, UZFG_UT002].*

*“Also, the parents, they have to be involved in the programmes so that they support their children, so that they don’t feel shy about it” [Male FGD, Young Adult, UZMG_Y006].*

Both the community members and HCPs felt that since men are key decision-makers in FP/C methods choices, they need to participate in FP/C activities so that they could better understand the benefits and therefore provide support to their female partners. They indicated that it was important for FP/C programmes to find innovative ways to involve men. Further, it was felt that both married and unmarried, as well as the sexually active and non-active community members should participate in FP/C programmes.

*“Both men and women should play an active part because if part of the community say ‘no this is for females alone’, then we will not win. But if everybody in the childbearing age plays an active part such that when they are given information, they share with a neighbour, then this information will go to the whole community and everyone will access family planning.” [Key stakeholder, Health sector, UZI003].*

The study participants narrated that incorporating community feedback in FP/C services programming, using existing mechanisms, would facilitate participation. The HCPs reported that appropriate use of complaint/suggestion boxes in healthcare facilities by community members could be one such mechanism of providing feedback on FP/C services. Complaint /suggestion boxes provide a platform to get community member’s views on their experiences with the quality of FP/C services. However, the HCPs indicated that there was limited information in most communities on the role of complaint/suggestion boxes. They stated that in most instances, community members complained directly to government officials or the media rather than engaging with the health facilities.

*“You can get information from the community’s responses to the services that are provided through the suggestion box as one of the ways. Through the suggestion box, they can even suggest on how best the FP/C services can be rebuilt in a health facility where they seeing some lapses. However, community members don’t know how to use these facilities well. Sometimes you only hear of these complaints in the media or from the politicians” [Healthcare Provider FGD, Managerial, UZHG_H004].*

Although suggestion/complaint boxes were widely available in all facilities, the HCPs indicated that community members were not using them. Community members believed that by using the suggestion/complaint boxes they would be risking their access to healthcare, including FP/C services if identified by the HCPs. The HCPs suggested that community health workers (CHWs) be used to educate community members on the importance of these suggestion/complaint boxes. They also recommended that the boxes should be located in convenient places where potential FP/C services clients could drop their complaints/suggestions without fear of possible discrimination or stigmatization.

*“Currently, suggestion boxes are not being utilized as they are supposed to be, because community members usually fear to be seen. They think maybe they might be stigmatized once they are seen going near that box to put in whatever suggestion they may have.” [Healthcare Provider FGD, Managerial, UZHG_H009]*

2. Engagement of existing community resources and structures

The second thematic category underscores the significance of utilising local resources, such as influential community leaders and community-based health structures and providers. Involving influential community persons, such as councillors, ward committee chairmen, religious leaders, headmen and chiefs, in community FP/C services provision efforts would facilitate community participation as these are gatekeepers who commanded respect in society. Some of these leaders had already been selected as FP champions to lead community mobilisation efforts in previous programmes. It was indicated that champions could use their political, traditional and religious influence to increase awareness on the importance and benefits of participating in FP/C services programmes.

*“I think if we can involve the leaders, we bring them on board and teach them the importance [of] family planning, it will help us a lot. You can imagine how many people are staying in [name of area], but there is headman there, there is a leader there, who can influence those men and make them understand the importance of family planning.” [Healthcare Provider FGD, Frontline, UZHG_L004].*

Both the HCPs and community members, particularly those from rural areas, indicated that community participation could also be facilitated through exploring and strengthening existing community health governance systems. Examples of these structures include Neighbourhood Health Committees and community-based health workers such as Traditional Birth Attendants, Safe Motherhood Action Groups and Community-Based Distributors. These community-based health structures and providers act as a link between the community and healthcare system, providing a wide variety of healthcare services. They conduct health education as well as provide FP/C methods such as oral contraceptive pills and condoms to communities. The HCPs stated that it was easier to work with the CHWs because of the already established relationships with the community members.

*“In this community, we have what we call the Safe Motherhood Action Groups, these usually disseminate information within our community, but for Kabwe I think the only people that are used to provide that information are the Community Based Distributors. Those are the ones that disseminate part of the information on family planning and also distribute basic family planning commodities.” [IDI, Key stakeholder, Health Sector, UZ1006].*

*“The community health workers are motivated when they are called for a meeting, and then they are given books, ball pen or maybe a bag with an ID. It is a lot of motivation to them. Others feel like bicycles are more motivation to them so that they are able to reach other families that are far away and provide health education [to] them or maybe even give contraceptives.” [Key stakeholder IDI, Health sector, UZI006].*

To further promote participation of local healthcare providers, community members suggested that capacity building be conducted among volunteers in providing contraceptive injections as well as FP/C counselling services to supplement the HCPs. Volunteers should not only possess counselling skills, but also the right approach technics to engage all households, including those who oppose use of FP/C methods in rural areas. Further, it was recommended that these counselling and outreach activities needed to build the capacity of households to discuss FP/C freely.

*“Even before we access the family planning from the clinic, even in our very homes we are coming from, we should try to talk to our children about the benefits of family planning since we know it already as parents that they are sexually active... I think community volunteers should encourage this practice in their outreach activities.” [Male FGD, Young Adult, UZFG_UZMG_Y003].*

The community members reported that discontinuation of most partner funded participatory health programmes was a challenge to participation. Some community members were said to be demotivated to get involved in new programmes as they had concerns that they may eventually close down. They reported that this often occurred in past participatory activities such as sport, drama and community groups. The community members recommended there was a need to motivate community members for them to continuously participate in FP/C services activities.

*“What I can say is that all the clubs, community groupings, the drama clubs and even sport, they can work and again they can’t work. What makes them work is the motivation. Wherever you go, when there is no motivation, nothing works, and where there is motivation things [go] well.” [Male FGD, Urban Young Adult, UZMG_Y001]*

3. Building trust in family planning and contraceptive methods and services

The third thematic category emphasises the role of building trust in FP/C methods/services to enhance community participation. One of the key challenges is related to addressing the associated myths and side effects of FP/C methods. Community members thought enhancing community participation means improving trust in FP/C methods and services by developing and disseminating appropriate information, community dialogues, as well as community participation in selecting community-based distributors. Community members reported that the promotion of specific FP/C methods to suit different user needs was cardinal in facilitating participation. They suggested that diffusion of information among community groupings such as local support and women groups was vital in building trust in FP/C services among social networks. It was also indicated that there was a need to ensure that appropriate information was developed through joint planning and information dissemination with the church.

*“I think maybe they should be people from the church and people from the health sector and again from the community so that when these people [sit] together, they can plan well, if there is a person who has to talk about natural family planning, they should talk within the same fora.” [IDI, Key stakeholder, Community leader, UZI009].*

The key stakeholders further indicated that participation and trust in FP/C services could be enhanced if the HCPs engaged community members in service provision using platforms such as community dialogues/meetings. The community members narrated that dialogue between the community and HCPs regarding adolescent use of FP/C methods would encourage parents to begin opening up to adolescent use. Further, these dialogues were also said to allow community members and HCPs to jointly develop solutions and address issues affecting FP/C services, hence foment participation and trust.

*“Continuous dialogue, training and meetings and other interactions between health workers and the community will make people aware of the benefits of family planning and participate in these programmes. It [is] also a nice platform for people to talk about some of the issues they have with family planning [Male FGD, Urban Male Adolescent UZMG_A003].*

Engagement of the community leaders in selecting appropriate community-based distributors (CBDs) was another strategy for building trust as it promotes the credibility of the CBDs. It was suggested that the selection of CBDs be done with the approval of the local leadership and general community membership to legitimise their work and enhance trust in the FP/C services. This locally-driven process assured community buy-in and support for FP/C services.

*“Concerns are there sometimes because of their status, especially where we don’t call the leader to explain to them that these are the people who we will be working with and will be in the community. The leaders will reject them because they have never been chosen by the leaders,” [Healthcare Provider FGD, Frontline, UZHG_L002]*

4. Facilitative strategies in family planning and contraceptive services

The fourth thematic category underscores community experiences with the status-quo and how it affects participation in FP/C services programmes. It highlights critical challenges to participation inherent within the design of health system structures for FP/C services and the possible role of facilitative strategies. The participants discussed various strategies, including defeminisation of FP/C services and making the health facilities responsive to the delivery of adolescent FP/C services. The key health sector stakeholders narrated that FP/C services were predominantly designed for women, which consequently excluded men from participating. This was visible in the terminologies used to refer to certain FP/C services, which seemed to imply that they are only concerned with women. For example, the terms “Maternal Child Health” or “Prenatal Services” were said to be designed without men in mind. According to the key stakeholders, embracing inclusivity would require revisiting the naming of certain FP/C services as well as creating the infrastructure that is welcoming to both males and females within the health system.

*“When you say parental, even I as a man I will feel welcome at this place. But if you are telling me this is a clinic for mothers and children you have excluded the men. You even go to the extent of creating a Ministry of Gender where you are excluding them [male].” [IDI, Key stakeholder, Health sector, UZI007].*

Supporting youth-friendly corners was cited as another key facilitative mechanism. Most of the HCPs reported that the youth-friendly corners at their health facilities were not fully functional. Not many youth were utilising FP/C services due to logistical and local challenges such as stigma, lack of privacy and confidentiality. Strengthening youth-friendly corners would provide a platform for HCP and adolescent engagement, as well as peer-to-peer learning mechanisms in FP/C services programming. Further, it was reported that providing adequate logistical support to youth-friendly corners would also encourage youth participation.

*“I think in the past we have somehow overlooked the teenagers, but there is now more emphasis on teenagers. The last time we had a planning meeting, we only had 12 youth-friendly corners in the whole province and some of them are not fully functional. There is [a] need to increase the number of and improve support to youth-friendly corners in all the facilities if we are to improve adolescent use of contraceptives” [Key stakeholder, Health sector, UZI006].*

## Discussion

Community participation provides an important platform for improving SRH outcomes, yet evidence of how it can be fostered in FP/C services provision programming remains inadequate. This study posits that facilitating community participation in FP/C services provision will require a focused approach that pays attention to four thematic areas of accountability, community engagement, building trust and fostering facilitative strategies in FP/C services programming. Though these findings were used to develop the UPTAKE intervention, they are applicable to other participatory programmes in similar contexts.

According to the WHO, ensuring accountability should be at the core of SRH programming [22]. The study participants narrated that accountability should include clarity about the responsibility of those implementing FP/C programmes in ensuring recruitment of appropriate community members, as well as enabling them to hold HCPs accountable for the quality of FP/C services, to give legitimacy to the participatory process and ensure support. This entails strengthening feedback and monitoring mechanisms, and ensuring that they are incorporated into FP/C services quality improvement efforts. Current feedback and monitoring mechanisms such as the suggestion boxes were said to be ineffective and fall short of public confidence. This may imply that community members are not satisfied with the actions taken on their complaints and suggestions, but also that they may not be aware of some of these mechanisms. More sensitization and engagement may be required.

The study participants highlighted the role of community dialogues/meetings, as well as, the credibility of the CBDs in building trust in FP/C methods/services. Trust underpins the co-operation between health providers and community members in FP/C services provision [23]. It originates and is sustained through interactions, relationships and social capital within FP/C services programmes. On the contrary, distrust in FP/C methods/services is fomented by negative experiences, such as the side effects [24]. Building trusting relationships between frontline providers and clients, and using a shared decision-making approach, as well as providing counseling about side effects, have been found to promote continuation and adherence to FP/C methods [25]. Other studies have equally reported that person-centered interventions focusing on trust have high success in improving perceptions of quality and knowledge of FP/Cs services among clients [24].

Shared decision-making approaches such as the community dialogues/meetings not only provide a platform for community and health provider interactions for building trust but can also be used as accountability mechanisms to design and monitor interventions in FP/C services. In this regard, the participants reported that dialogue between the community and health providers would help identify obstacles affecting the use of FP/C services, and allow for the joint development of locally relevant implementable solutions. Other studies have used community dialogues in identifying and implementing context-relevant interventions to improve maternal and child health outcomes [26]. Furthermore, community dialogues can facilitate participation in health services by providing a platform for the health system to engage community actors such as parents and religious leaders who are vital in generating wider community support FP/C services [27].

Acknowledging the role of CHWs and strengthening their capacity to deliver FP/C methods is critical to improving support for services. However, the limited range of FP/C methods provided by the CHWs was said to limit the options for clients and potential users. The participants indicated that there was a need to build capacity in CHWs to provide injectable contraceptives as this was their preferred method. While the standard practice in Zambia is that injectable contraceptives are provided by qualified health providers, other studies have reported that it is possible to train the CHWs. A study from Ethiopia found that receiving injectable contraceptives from CHWs proved to be as safe and acceptable as from the health workers at the facility [28]. Training of CHWs to provide injectable contraceptives may perhaps be an option for the Zambian government. However, this should be accompanied by clear guidelines and monitoring.

Fostering facilitative strategies that look to address structural failures such as feminisation of FP/C services to create an enabling environment for male involvement in FP/C services. Other studies have documented the dominant role of male partners in household FP/C decision-making in Zambia [29]. However, the findings indicate that study participants found FP/C services are generally unwelcoming to men, which has also been reported elsewhere [30, 31]. Facilitative strategies may seek to revisit the naming of some FP/C services to be more male inclusive. Improving accessibility and accommodation of FP/C service venues may make them more attractive for male partners [32]. Interventions that encourage couple FP/Cs discussions have yielded positive results in improving husband approval and couples uptake of FP/C [33, 34]. In addition, interventions targeting male involvement should not bypass efforts to empower women by reinforcing gender inequities, rather than challenging them [35, 36].

Facilitative strategies also entail making the health facilities responsive towards the delivery of adolescent FP/C services. The HCPs indicated the need for more investments to support youth-friendly corners. However, the effectiveness of youth-friendly corners in addressing adolescent FP/C services needs remains unclear in the literature. A systematic review on the effectiveness of youth centers in increasing use of SRH services in low and middle-income countries found that uptake of services was generally low despite the widespread emphasis on youth centers [37]. Potential methods to increase adolescent uptake may include the linking of school education programmes with youth-friendly services, life skills approaches, and social marketing and franchising [27]. Another study suggests the need for investment in strategies that aim to create a more supportive environment for adolescent participation in FP/C services [38].

While community participation is undoubtedly a key component of delivering inclusive and effective FP/C services, challenges regarding how best it can be achieved, integrated and measured remain. Participation outcomes are measured not by the levels of healthcare utilisation, but by the processes by which communities are involved and own the implementation process of FP/C programmes. Given the conceptual and technical issues, the findings from this study provide important evidence for consideration in designing and implementing successful participatory programmes. Those implementing similar FP/C services programmes should, therefore, reflect on the highlighted factors, and seek to incorporate them into their programming.

### Strengths and limitations

Conducting the study in only one setting in Zambia, the use of a small, purposively selected sample of respondents, as well as using only a qualitative approach to collect data, limits the extent to which the study can be generalized. However, generalizability was not the intention of this study. The rich description of phenomena (community participation) has helped in developing an account that we believe provides a valuable contribution to the knowledge on FP/C services provision and use in low-income settings. The collection of data from the various categories of participants enabled the gathering of a wide range of views on key issues affecting community participation in FP/C services programmes at different levels, which allowed for strengthened triangulation of perspectives on key thematic areas. Furthermore, the iterative reading of the collected data allowed for constant comparisons to increase the validity of the emergent themes.

## Conclusions

Community participation interventions that are context-specific, involving HCPs and community members can play a significant role in increasing met needs and also sustaining FP/C use. However, achieving meaningful participation from both community and HCPs remains a challenge. Attention should be paid to identifying and recruiting the right community participants who represent and have a vast understanding of community FP/C needs. There is a need to address the power relations within the community, as well as, between HCPs and the community. This study posits that facilitating community participation in FP/C services provision will require a focused approach that pays particular attention to the four thematic areas of accountability, community engagement, building trust and fostering facilitative strategies in FP/C services programming. Understanding and considering community members and HCP’s views regarding locally relevant participatory approaches, facilitators and challenges to participation, could improve the design, implementation and success of participatory public health programmes, including FP/Cs. Future research may, therefore, focus on understanding the identified thematic areas that facilitate participation, and how they produce change in FP/C services provision through a process evaluation.

## Data Availability

The data are not publicly available as it contains information that could compromise research participant privacy/consent. However, some anonymised aspects of the datasets may be available upon request and with permission of the Department of Sexual and Reproductive Health and Research, World Health Organisation. Note that data sharing is subject to WHO data sharing policies and data use agreements with the participating research centres.

## Abbreviations

CBDs: Community-based distributors
CHWs: Community health workers
FGDs: Focus group discussions
FP/C: Family planning/contraceptive
HCPs: Healthcare providers
IDIs: In-depth interviews
NGO: Non-governmental organisation
NHC: Neighbourhood health committee
SMAGs: Safe motherhood action groups
SRH: Sexual and reproductive health
STIs: Sexually transmitted infections
TBAs: Traditional birth attendants
WHO: World Health Organisation
